# Classification and regression tree (CART) model to predict pulmonary tuberculosis in hospitalized patients

**DOI:** 10.1186/1471-2466-12-40

**Published:** 2012-08-07

**Authors:** Fabio S Aguiar, Luciana L Almeida, Antonio Ruffino-Netto, Afranio Lineu Kritski, Fernanda CQ Mello, Guilherme L Werneck

**Affiliations:** 1 Instituto de Doenças do Tórax (IDT)/Clementino Fraga Filho Hospital (CFFH), Federal University of Rio de Janeiro, Rua Professor Rodolpho Paulo Rocco, n° 255 - 6° Andar - Cidade Universitária - Ilha do Fundão, 21941-913, Rio de Janeiro, Brazil; 2 Harbor Hospital, 3001 S. Hanover St, Baltimore, MD, 21225, USA; 3 Ribeirão Preto Medical School, University of São Paulo, Av. Bandeirantes, 3900, 14049-900, Ribeirão Preto-SP, Brazil; 4 Instituto de Estudos em Saúde Coletiva, Federal University of Rio de Janeiro, Praça Jorge Machado Moreira, Ilha do Fundão, Cidade Universitária, 21944-210, Rio de Janeiro, Brazil; 5 Instituto de Medicina Social, State University of Rio de Janeiro, Rua São Francisco Xavier, 524, 7° andar, Bloco D. – Maracanã, 20550-900, Rio de Janeiro, Brazil

**Keywords:** Sensitivity and specificity, Accuracy, Tuberculosis, Diagnosis, Predictive models, CART

## Abstract

**Background:**

Tuberculosis (TB) remains a public health issue worldwide. The lack of specific clinical symptoms to diagnose TB makes the correct decision to admit patients to respiratory isolation a difficult task for the clinician. Isolation of patients without the disease is common and increases health costs. Decision models for the diagnosis of TB in patients attending hospitals can increase the quality of care and decrease costs, without the risk of hospital transmission. We present a predictive model for predicting pulmonary TB in hospitalized patients in a high prevalence area in order to contribute to a more rational use of isolation rooms without increasing the risk of transmission.

**Methods:**

Cross sectional study of patients admitted to CFFH from March 2003 to December 2004. A classification and regression tree (CART) model was generated and validated. The area under the ROC curve (AUC), sensitivity, specificity, positive and negative predictive values were used to evaluate the performance of model. Validation of the model was performed with a different sample of patients admitted to the same hospital from January to December 2005.

**Results:**

We studied 290 patients admitted with clinical suspicion of TB. Diagnosis was confirmed in 26.5% of them. Pulmonary TB was present in 83.7% of the patients with TB (62.3% with positive sputum smear) and HIV/AIDS was present in 56.9% of patients. The validated CART model showed sensitivity, specificity, positive predictive value and negative predictive value of 60.00%, 76.16%, 33.33%, and 90.55%, respectively. The AUC was 79.70%.

**Conclusions:**

The CART model developed for these hospitalized patients with clinical suspicion of TB had fair to good predictive performance for pulmonary TB. The most important variable for prediction of TB diagnosis was chest radiograph results. Prospective validation is still necessary, but our model offer an alternative for decision making in whether to isolate patients with clinical suspicion of TB in tertiary health facilities in countries with limited resources.

## Background

Even after 50 years of effective treatment, tuberculosis (TB) remains a major public health issue worldwide. Its airborne transmission endangers all individuals irrespective of social class or country of origin, although it affects mostly the poorer groups of the society [1]. For disease control to be achieved, prompt diagnosis and effective treatment of active cases, associated with treatment of latent TB infection (LTBI) are essential [2]. Sadly, most of the modern diagnostic tests have not yet become available in resource constrained countries [3], which concentrate 95% of world’s TB cases and 98% of deaths [1]. In these settings, diagnosis is still dependent of detection of acid-fast bacilli (AFB) through sputum smear analysis (SSA) or isolation of *Mycobacterium tuberculosis* (Mtb) after growth in solid culture medium. Although inexpensive and widely available, SSA has a low sensitivity and, in areas with laboratory shortage, results can take up to 7 days instead of a few hours [2].

The lack of specific clinical symptoms to predict pulmonary TB diagnosis makes the correct decision to admit patients to respiratory isolation (RI) a difficult task for the clinician. Since rapid RI of suspected cases is highly effective in preventing hospital transmission [4-7], overuse of isolation rooms (IR) is common, with described rates of TB diagnosis ranging from 3.7 to 44% among patients admitted to RI [8-14]. As a consequence, medical costs are increased due to the need for installing and maintaining IR [15-17]. IR are still scarce and infection control measures in health care facilities are at an early stage of development in most resource constrained countries, as shown by recent data from the WHO [18].

For more than 10 years it has been hypothesized that the identification of clinical parameters readily available at the time of admission can improve the use of isolation rooms [8]. Predictive models need to be validated in the population where it will be applied, since even high accuracy models might perform poorly in a population with different TB epidemiology [19]. As a consequence, no prediction models have been validated for use in multiple settings.

Brazil ranks 14th in the World Health Organization (WHO) list of countries with highest burden of the disease [18]. Rio de Janeiro City (RJC) has a incidence of TB of around 105.5 cases per 100,000 habitants [20] and one third of its cases are diagnosed in hospitals [21]. Such environments have an established role in the transmission of TB and, although infection control measures are considered by the WHO as essential [18], few hospitals in RJC have implemented any of these measures. As a consequence, an increased risk of transmission to other patients and health care personnel is expected in these settings as shown in data from developed countries and South Africa [7,22-25]. Recently, outbreaks of XDR-TB have been reported in hospitals from South Africa [25].

The decision to isolate patients is largely based in physician experience and intuition but this can be misleading [26]. Clinical prediction rules have been developed to assist the clinician in decision making of isolation, with utilization of many statistical techniques such as logistic regression and neural networks, for example [27]. Few studies used CART methodology to predict TB diagnosis [8,28,29], two of which have been performed by our group among outpatients in RJC. Mello et al [28] have applied CART to identify patients with smear-negative pulmonary tuberculosis (SNTB) with good results. Santos et al [29] also showed good results in applying CART to SNTB patients. To our knowledge the only study that applied CART methodology to predict TB in hospitalized patients was performed by El-Sohl et al [8] in the USA. The researchers described a simple model able to reduce unnecessary RI by 40%.

Clinical algorithms can increase the pretest likelihood of TB diagnosis in high and low income countries [30]. Since substantial economic costs are related to unnecessary isolation of patients [31], a clinical model to predict active TB in patients admitted to hospitals can become an important tool for improving infection control in resource constrained countries with high disease burden. The use of such models at the moment of arrival at the health unit may be able to lower utilization of IR in patients with other diseases, thus reducing costs and improving the rationale utilization of such beds [9]. Therefore, we studied a hospitalized sample of patients in a tertiary hospital located in a high TB prevalence area of RJC to develop a predictive model for pulmonary TB aiming at contributing to a more rational decision on the use of isolation rooms (IR).

## Methods

We performed a cross sectional study among patients admitted to IR of the Clementino Fraga Filho Hospital (CFFH) of the Federal University of Rio de Janeiro. CFFH is a tertiary hospital, reference for the treatment of patients with HIV/AIDS. In 1998, a TB control program (TCP) was implemented in CFFH as a novel strategy for the control of TB with significant reduction of LTBI in health care workers (HCW) [32]. The program consisted of isolation of TB suspects and confirmed TB inpatients, quick turnaround for acid-fast bacilli sputum tests and HCW education in use of protective respirators, with a consistent reduction in tuberculin skin test conversion among HCW.

From March 2003 to December 2004, a convenience sample of all patients admitted in IR had their medical charts reviewed. Inclusion criterion was clinical suspicion of TB. We defined clinical suspicion in the same way patients in our hospital are selected for RI: cough for more than 2 weeks associated with any radiologic abnormality, or any respiratory symptom in patients with confirmed or suspected HIV infection. Patients with active TB diagnosis previous to the admission, with extra-pulmonary TB and without a final diagnosis were excluded. Decision to isolate these patients was made by emergency room or TB control program physicians according to the TB program criteria. Patients HIV negative with cough for more than 2 weeks and an associated abnormality in a chest X-ray were considered suspect of having TB and were isolated. For patients HIV positive were considered suspects if they had any respiratory symptom and were also isolated. Clinical data regarding demographic characteristics, respiratory and constitutional symptoms, potential predictive factors for TB diagnosis, radiologic test results and final diagnosis of admission were analyzed retrospectively. Radiologic tests were analyzed in a standard manner by a pulmonologist (*L.L.A.*) with experience in TB care, blinded to patient’s information. The tests were classified as either normal or sequelae from a previous TB episode, suggestive of TB by typical or possible X-ray findings and atypical findings (Table 1). Typical were those considered as having any parenchymal infiltrate or cavity localized in the upper zone (defined as the area above the posterior third rib); possible were those presenting a miliary pattern, pleural effusion or thoracic adenopathy, and atypical those showing any other abnormality.

**Table 1:** Description of x-ray findings

**X-ray finding**	**Description**
Suggestive	Infiltrate or cavities in one of more segments of superior lobes and/or superior segments of lower pulmonary lobes, miliary pattern, pleural effusion and/or thoracic adenopathy
Normal or Sequelae	Normal X-ray or findings suggestive of a previous TB episode, without suspicion of active disease
Atypical	Any abnormalities not classified by Suggestive or Possible

Pulmonary TB was defined by isolation of *Mycobacterium tuberculosis* (Mtb) in *Lowesten-Jensen* (L-J) solid culture medium in respiratory samples (spontaneous or induced sputum and bronchoalveolar lavage), by findings of granulomatous inflammation with caseous necrosis in respiratory tissue biopsy samples or by improvement of respiratory symptoms within 60 days of TB treatment, without treatment for other diseases. A minimum of two spontaneous sputum samples were analyzed for each patient. Those without sputum were submitted to one sample of induced sputum or bronchoscopy with bronchoalveolar lavage for analysis.

Differences in the prevalence of pulmonary TB by potential predictors were analyzed using the Chi-Square test for categorical variables or the Mann-Whitney test for continuous variables. Associations between putative predictive factors and the outcome were expressed as odds ratio (OR) and their respective 95% confidence intervals (95%CI) estimated by logistic regression.

We developed a CART model using S-Plus 4.5 (MathSoft, Inc) software. CART builds a tree through recursive partitioning, so the data set is successfully split into increasingly homogenous subgroups. At each stage (node) the CART algorithm selects the explanatory variable and splitting value that gives the best discrimination between two outcome classes. A full CART algorithm adds nodes until they are homogenous or contains few observations (≥5 is the standard cut off in S-Plus). The problem of creating a useful tree is to find suitable guidelines to prune the tree. The general principle of pruning is that the tree of best size would have the lowest misclassification rate for an individual not included in the original data [33].

Data collected from all patients were included in the model. Patients with missing HIV serology results were joined with the HIV negative group (HIV negative/undeterminate), since patients with clinical suspicion of HIV infection were more likely to have a test requested by the attending physician. The predictive variables included in the model were chest X-Ray results (as described in Table 1), age, gender, cough for more than 3 weeks, HIV/AIDS, hemoptysis, weight loss >10% of body weight, dyspnea, fever, smoking and alcohol use history and recent contact with a pulmonary TB case. The response variable was final diagnosis of pulmonary TB. The process of growing the tree was stopped when we found a gain of less than 1% of the classification error or when the number of patients within each knot was less than five. We then validated the model with another convenience sample of patients with similar characteristics admitted to IR of the hospital in a one year period from January to December 2005. This sample consisted of 191 individuals admitted to the hospital with clinical suspicion of pulmonary TB from January to December 2005. The prevalence of TB in the validation sample was 16.6%. HIV prevalence was 46.6%. Other clinical and radiological characteristics were similar to the original sample.

The area under the ROC curve, sensitivity, specificity, positive and negative predictive values with their respective 95% confidence intervals, estimated using Stata software, version 9.0, were used to evaluate the performance of the model. The study was approved by the CFFH Ethics Committee.

## Results

From March 2003 to December 2004, 315 patients were admitted to RI in CFFH with clinical suspicion of TB. We excluded 25 patients due to TB diagnosis previous to the admission (n = 15) and absence of final diagnosis (n = 10). Data was analyzed for the remaining 290 patients. Pulmonary TB diagnosis was confirmed in 26.5% (77/290) of the patients, with isolation of Mtb in 72 patients (48 had positive SSA). In addition, 2 had chronic granulomatous inflammation with caseous necrosis and three had TB confirmation by clinical improvement with TB treatment. SNTB was present in 37.7% of pulmonary TB cases. HIV/AIDS was present in 56.9% (n = 165) of patients. In the HIV group, SNTB was present in 48.6% (n = 82). Three HIV positive patients with AFB in SSA had identification of non-tuberculous mycobacteria (NTM) (5.9% of positive SSA). Medium age was 42 years. Other clinical, demographic and radiologic data of the patients are displayed in Table 2.

**Table 2:** Clinical and radiologic characteristics of the patients included and associations with pulmonary TB

		**N**	**(%)**	**OR**	**95% CI**
*Demographic Data*					
Gender					
	Male	173	59.7	1	
	Female	117	40.3	0.53	0.30-0.94
Age					
	Mean	43.2	41.48 – 44.93		
	< 31 yrs	60	20.7	1	
	31-40 yrs	73	25.2	0.48	0.22 – 1.04
	41-50 yrs	66	22.7	0.55	0.25 – 1.19
	51-60 yrs	53	18.3	0.56	0.24 – 1.27
	>60 yrs	38	13.1	0.61	0.25 – 1.50
HIV/AIDS					
	No/ Undeterminate	125	43.1	1	
	Yes	165	56.9	0.61	0.36-1.04
*Clinical Characteristics*					
Fever					
	No	85	29.3	1	
	Yes	205	70.7	1.37	0.76-2.48
Cough for more than 3 weeks					
	No	162	58.3	1	
	Yes	116	41.7	2.49	1.44-4.28
Hemoptysis					
	No	235	81.0	1	
	Yes	55	19.0	0.48	0.22-1.03
Weight Loss					
	No	165	56.9	1	
	Yes	125	43.1	3.40	1.97-5.88
Dyspnea					
	No	168	57.9	1	
	Yes	122	42.1	0.62	0.36-1.07
Recent Contact with TB					
	No	262	91.3	1	
	Yes	25	8.7	1.64	0.69-3.87
*Habits*					
Smoking History					
	No	134	47.7	1	
	Yes	147	52.3	1.22	0.72-2.08
Alcoholism					
	No	241	84.0	1	
	Yes	46	16.0	0.89	0.42-1.85
*Radiological Results*					
Chest X-Ray					
	Normal or Sequelae	89	30.7	1	
	Typical or Compatible	81	27.9	68.08	19.58 – 236.69
	Atypical	120	41.4	4.73	1.34 – 16.68

**Figure 1:**
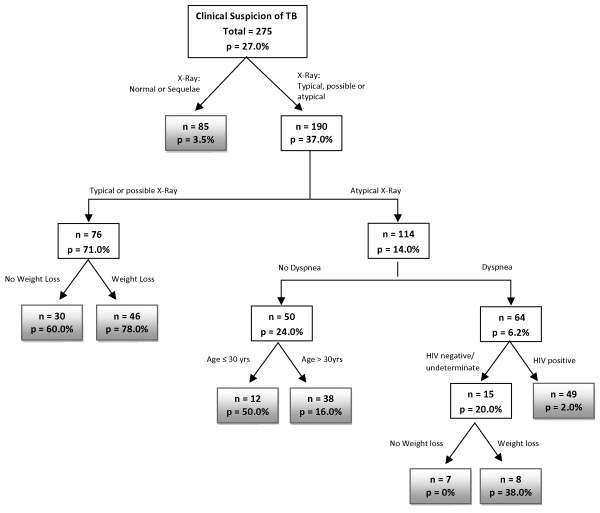
**Classification and regression tree model for predicting pulmonary tuberculosis (TB) in hospitalized patients.** The number of patients (n) and the probability of TB (p) are given inside each node. Terminal nodes are shaded.

The generated CART model is displayed in Figure 1. Only 275 patients were included in this model due to missing values in one or more variables. The variable with the greatest discriminative power was the x-ray result. The validated CART model showed sensitivity, specificity, positive predictive value and negative predictive value of 60%, 76%, 33%, and 90%, respectively. The AUC was 79% (Table 3). The minimum number of patients in the parent and daughter nodes were 15 and 7, respectively. The residual mean deviance was 0.108 and the misclassification rate was 15%.

**Table 3:** Results from validation of the CART model – Sensitivity, Specificity, Positive and Negative predictive values and area under the ROC curve

	**%**	**95% CI**
Sensitivity	60	40 – 77
Specificity	76	68 – 82
Positive Predictive Value	33	21 – 47
Negative Predictive Value	90	84 – 95
Area under the ROC curve	79	70 – 88

## Discussion

The CART model developed for these hospitalized patients with clinical suspicion of TB had fair to good accuracy for pulmonary TB as indicated by the area under the ROC curve. The model was developed to achieve a high specificity in order to avoid nosocomial transmission, but had also fair to high sensitivity. The sensitivity of the model is higher than the sensitivity of sputum microscopy examination among all suspected cases (48%) and in HIV patients (30%) [32]. This result is relevant since it is common in some settings, mainly in resource constrained countries, to have smear examination for acid fast bacilli as the only test available for pulmonary TB diagnosis. The model also had a high negative predictive value (90.55; 95% CI 84.08–95.02).

In our sample of all patients submitted to RI, only 26.6% had TB confirmed. The high negative predictive value found in the CART model allows its application in patients with clinical suspicion of TB in the emergency room in order to lower the number of unnecessary RI. The application of a predictive model in patients with clinical suspicion of TB has been described before and was able to reduce the number of unnecessary isolations without increasing the risk of nosocomial transmission [14].

Our model had a high negative predictive value, similar to the CART model described by El-Sohl et al [8], with an overall higher accuracy. We also had a higher accuracy than the CART model developed by Mello et al in RJC that included only SNTB [28]. This finding is expected since SNTB is a factor known to harden TB diagnosis [28]. Two studies have used neural networks for case detection in hospitalized patients. El-Sohl et al [9] described a model with a sensitivity of 92.3% and specificity of 71.6% for case detection, which are higher than we found in our model. Santos et al, studying SNTB, constructed a neural network with accuracy similar to ours, being able of correctly classifying 77% of the cases [29].

The most important variable for prediction of TB diagnosis was chest radiograph results. Typical or compatible x-rays were found to predict the diagnosis of pulmonary TB. This finding has been previously reported in CART models for TB in hospitalized patients [8]. Although chest radiography has been described as less specific for TB diagnosis and with a higher cost for case detection in outpatients with clinical suspicion of TB [34], for hospitalized patients the test seems to have clinical importance. Age has been described as important for prediction of TB in RJC patients [28,29]. In our model, a cutoff of 30 years of age was important for discriminating TB, particularly in patients with atypical chest X-Ray without dyspnea.

Predictive models for the diagnosis of TB provide a useful framework for systematization of the diagnostic approach [35] and are able to standardize data collection from clinicians [36], optimize high cost resources such as IR [29] and lower empiric treatments. In order to achieve control of TB new low-cost, highly accurate tests, are essential for use in areas with high TB prevalence. CART methods build a binary classification system according to the variable with the greatest capacity for discriminating between outcomes (in this case, the presence or absence of TB). The discriminatory power decreases with each subsequent division. The main advantages of CART are that it is simple, interactions between the variables can be identified directly from the model and probability can be displayed in the tree. Its simple structure makes it easy for the clinician to understand the data displayed, unlike some other statistical methods. It is also inexpensive and allows immediate results. Therefore, it can become a tool for TB diagnosis in resource limited settings.

Predictive models should be applied to populations where they were validated [27,28]. Our model was validated with the use of a sample of different patients admitted to the same hospital in another period of time, reason why we assume it to be a robust model for prediction of TB diagnosis. The main strength of our model is to allow utilization in resource limited settings since it has been developed from individuals attending a health unit in a city with high prevalence of TB and with a number of restrictions in the availability of diagnostic resources for TB. The variables selected can be easily obtained by clinical interview and a chest radiograph, allowing its use for rapid isolation decision. Also, the high accuracy of the model allow prompt use in a population of hospitalized patients, a population known to be difficult to diagnose TB due to the high number of alternative diagnoses, especially in HIV/AIDS patients.

Our study has some limitations. All data was collected retrospectively, increasing the risk of information bias due to risk of incomplete registry of data and potentially increasing the accuracy [37]. This limitation is inherent to the development of such models, and further validation in prospective studies is necessary. Also, since we studied a convenience sample of patients admitted to RI, this might not be representative of the population we wish to make inferences and might also not meet the sample size requirements for generating models with the best possible predictive performance. Another limitation is that the model was generated with data from patients admitted in a tertiary hospital, limiting the generalization of the results. Selection bias is another potential problem, since we studied a convenience sample and not a probabilistic sample, and it is possible that the studied population does not represent the target population for whom we wanted to make inferences. Also, patients were selected after admission to an isolation room, thus increasing the pretest probability of TB. Our main discriminative variable was chest radiography. Other studies have a different classification of thoracic radiography [8-13,26,27]. To our knowledge, there is no data in the literature to define a universal classification system. We used the same classification method from previous studies of predictive models from our group in order to maintain standardization of our findings [28,29]. Last, the approach to classify patients with missing HIV serology results and low clinical suspicion for HIV infection in the HIV negative/undeterminate group may have misdiagnosed some of these patients, interfering with the accuracy of the model.

## Conclusion

Prospective validation is necessary, but our CART model offers an alternative for decision making in whether to isolate patients with clinical suspicion of TB in tertiary health facilities in countries with limited resources. A reasonable strategy for the present model would be its application in patients with clinical suspicion of TB who demand admission to a hospital with a limited number of IR, especially for HIV/AIDS patients. Patients with a low probability of TB in the model can have bacteriologic analysis while admitted in regular hospital beds, especially those with confirmed or suspicion of HIV/AIDS diagnosis. Nonetheless, currently there are no predictive models for this purpose that can be generalized for all settings. CART models are an alternative for the development of such clinical decision rules, but other statistical techniques, such as logistic regression and neural networks, are available and more studies are needed to define which would have the best performance for predicting TB and thus contribute to a more rational decision on the use of isolation rooms (IR). Further studies are needed with prospective data before these tools can become clinical practice in resource constrained countries with high TB prevalence.

## Competing interests

The author(s) declare that they have no competing interests.

## Authors’ contributions

FSA analyzed the data, constructed the model and wrote the final manuscript; LLA had the idea, wrote the study project, collected the data and performed the preliminary analysis, AR-N discussed and made changes to the study project, performed orientation during the data collection and preliminary analysis; ALK discussed and made changes to the study project, performed orientation during the data collection and preliminary analysis; FCQM discussed and made changes to the study project, performed orientation during the data collection and preliminary analysis; wrote the final manuscript; GLW wrote the methods section on the study project, constructed the model, validated the model and wrote the final manuscript. All authors read and approved the final manuscript.

## Pre-publication history

The pre-publication history for this paper can be accessed here:

http://www.biomedcentral.com/1471-2466/12/40/prepub
